# Molluscicidal effect of *Achyranthes aspera* L. (Amaranthaceae) aqueous extract on adult snails of *Biomphalaria pfeifferi* and *Lymnaea natalensis*

**DOI:** 10.1186/s40249-017-0349-4

**Published:** 2017-10-16

**Authors:** Belayhun Mandefro, Seid Tiku Mereta, Yinebeb Tariku, Argaw Ambelu

**Affiliations:** 1 Department of Environmental Health Science and Technology, College of Public Health, Jimma University, P.O. Box 378, Jimma, Ethiopia; 2 Department of Biology, College of Natural and Computational Sciences, Dilla University, P.O. Box 419, Dilla, Ethiopia; 3 Department of Chemistry, College of Natural Science, Jimma University, P.O. Box 378, Jimma, Ethiopia

**Keywords:** *Achyrantes aspera*, Ethiopia, Snail control, Plant molluscicides

## Abstract

**Background:**

Intestinal schistosomiasis caused by *Schistosoma mansoni* is a wide spread disease in most parts of Ethiopian highlands. Snail control is one major strategy in schistosomiasis control. The use of molluscicidal plant products is becoming interesting due to their environmental friendliness, accessibility and easy application. This research is aimed to evaluate the molluscicidal effect of *Achyranthes aspera* on *Biomphalaria pfeifferi* and *Lymnaea natalensis* snails, which are of great medical and veterinary importance in Ethiopia.

**Methods:**

Adult *B. pfeifferi* snails were exposed to the various concentrations of *A. aspera* aqueous leaf extract for 24, 48 and 72 h. Similarly, adult *L. natalensis* snails were exposed to the extract for 24 h. Mortality data were analyzed using probit regression model. Phytochemical content of the plant was analyzed using standard screening methods.

**Results:**

The plant’s molluscicidal effect on the two snail species was demonstrated. The 24 h LC_50_ and LC_90_ values against *L. natalensis* were 69.5 and 93.9 ppm respectively. In the 24, 48 and 72 h exposure of *B. pfeifferi*, the LC_50_ values were 72.4, 69.9, 64.7 ppm and the LC_90_ were 96.5, 93.8, 92.8 ppm, respectively. The phytochemical screening tests indicated presence of saponins.

**Conclusion:**

From the findings of this study, *A. aspera* has a molluscicidal potential. The result provides a useful foundation for further in-depth studies to ensure its wider applicability in different water bodies and evaluate its toxic effects on non-target species.

**Electronic supplementary material:**

The online version of this article (doi:10.1186/s40249-017-0349-4) contains supplementary material, which is available to authorized users.

## Multilingual abstract

Please see Additional file 1 for traslations of the abstract into the five official working languages of the United Nations.

## Background

Schistosomiasis is a widespread parasitic disease endemic to over 74 countries infecting more than 200 million people in Africa, South America and Asia [1–3]. About 85% of the infection is found in Sub-Saharan Africa [4]. In Ethiopia, the snail *Biomphalaria pfeifferi* is the principal intermediate host of *Schistosoma mansoni* [1, 5]. The other species, *Lymnaea natalensis* is responsible for *Fasciola*, which is of great veterinary importance. Intestinal schistosomiasis is prevalent in the country, especially in the range of 1300 to 2000 m altitudes [6].

In addition to chemotherapy, integrated snail control strategies are important measures in schistosomiasis control especially in low endemic areas [7]. Currently, synthetic molluscicides are disregarded due to their high cost and environmental issues [8]. Plant products are becoming more and more interesting alternatives for their wide range of ideal properties including biodegradability and target specificity. As a result, molluscicidal plant researches are becoming so popular that many plant species have been screened [8, 9]. According to recent studies, *Glinus lotoides* [7] and *Jatropha gossypiifolia* [10] were found to be effective molluscicidal plants. In spite of a high botanical biodiversity potential and a variety of traditionally claimed medicinal plants, such studies in Ethiopia are limited [11, 12].

*Achyranthes aspera* (Family Amaranthaceae) is locally known as “Telenge” or “Ambulale” in many parts of Ethiopia. It is an esteemed medicinal plant in Asia, South America and Africa [13]. In Ethiopian folklore, it is used to control fertility, placental retention, postpartum bleeding, for eruption treatments and wound dressing [14, 15]. Yet, the action of *A. aspera* on those medically important snails is not known so far. The objective of this study is, therefore, to investigate the molluscicidal property of this plant.

## Methods

This is an experimental study designed to investigate the molluscicidal effect of *A. aspera* aqueous leaf extract on *B. pfeifferi* and *L. natalensis* snails in the laboratory condition.

### Plant material collection and processing

The plant *A. aspera* is selected based on the result from a preliminary screening test carried out earlier by the same researchers. Mature green leaves were collected from a place located at 9°43′45.59″ N, 39°73′2.71″ E nearby Debre Berhan Town, central Ethiopia. An expert called Mr. Melaku Wondafrash carried out plant species identification at Addis Ababa University herbarium. Voucher specimen was kept in the herbarium with specimen number: M.B.1. Some 400 g of leaves were dried in the shade to stable weight and ground to fine powder of mesh size about 200 μm according to Ndamukong et al. [4]. This was kept in tight plastic bag labeled and stored in dry and cool place in the laboratory of Environmental Health Science and Technology, Jimma University, Ethiopia.

### Snail collection

Adult snails of *B. pfeifferi* and *L. natalensis* were collected from stream habitats located at 7°41′15.39″ N, 36°50′52.32″ E in Jimma prison agricultural field, southwestern Ethiopia. They were taken to the same laboratory in a clean bucket with some water from the stream and were acclimatized for 3 days at room temperature, 12 h light and 12 h dark photoperiod in aged tap water. They feed on par-boiled dried shred of lettuce (*Lactuca sativa*) leaf. In the course of the experiment, the snails were ethically handled in accordance with the principles of animal welfare in scientific experiments.

### Aqueous extract and stock solution preparation

Aqueous leaf extract of *A. aspera* was prepared based on Ndamukong et al. [4]. 1 g plant leaf powder was soaked in 800 ml aged tap water in a flat-bottomed airtight flask and shook for 24 h on an orbital shaker at 110 rpm. It was then reconstituted to 1000 ml and directly used as a stock solution of 1000 ppm as mentioned in Kiros et al. [7].

### Test on adult *L. natalensis* snails

Exactly 60 ml serial dilutions of 25, 50, 75, 100, 200, and 400 ppm were prepared from the stock solution, each in a clean petri dish. Ten adult snails, 9-11 mm shell height, were exposed to each dilution for 24 h at room temperature. The test was performed in three replicates together with negative controls consisting of only aged tap water. Positive control was prepared from a 0.28 ppm niclosamide (Bayer and Pro-Serv. Inc. Germany). This concentration was considered as the LC_50_ value from the researcher’s previous experiment on the same snail species.

### Test on adult *B. pfeifferi* snails

This experiment was arranged in to three sets of exposure time: 24, 48, and 72 h. Exactly 60 ml test solutions containing 25, 50, 75, 100, 200, and 400 ppm were prepared to each of the three sets. Ten adult snails, 8.5-11 mm shell diameter, were exposed to each dilution. Each set was prepared in three replicates together with controls. Negative control was made from aged water only and positive control was made from 0.28 ppm niclosamide (for 50% mortality).

### Snail death recording

Once snails are exposed to the treatments in relation to the respective exposure times, each group of snails were removed, washed with tap water, and kept in aged water with food for another 24 h. Finally, dead snails in each group were counted with thorough inspection. Snails were considered dead if they remain inactive when pocked with a needle or remain retracted in to the shell or else the color of the shell and foot changes to foggy white [4, 7].

### Phytochemical analysis

Phytochemical screening tests were done using standard procedures as described in Akinyemi et al. [16] as well as Aiyegoro and Okoh [17]. Reducing sugars were identified by Fehling’s test in which the filtrate was heated with Fehling’s reagent for 5-10 min in water bath. For protein identification, Biuret test was performed by adding 4% sodium hydroxide and 1% copper sulfate solution. In addition, Ninhydrin test was done by heating the extract with 5% Ninhydrin (in butanol) solution in water bath for 10 min. Saponins were identified by formation of persistent foam after vigorous shaking of the aqueous extract. Total phenolics and tannins were tested by 5% and 10% ferric chloride solutions, respectively. Test for flavonoids was by treating the extract with concentrated hydrochloric acid and few pellets of magnesium turning for appearance of tomato red color. On top of that, Wagner’s test was applied for alkaloids using solution of iodine in potassium iodide for a reddish-brown precipitate.

### Data analysis

Probit regression analysis in IBM SPSS software version 20 was used to determine the LC_50_ and LC_90_ values, which denote effective doses for 50% and 90% mortality respectively. These are the commonly used endpoints in modern dose-response experiments [18–20]. The associated Chi-square (*χ* ^2^) values were used to assess the Pearson’s goodness-of-fit for the appropriateness of probit model to this particular data [19].

## Results

When *B. pfeifferi* were exposed to the concentrations of 200 and 400 ppm, they stopped crawling and defecating within the first two or three hours. In the beginning, they profusely secrete mucus and later start bleeding. *L. natalensis* snails produce less mucus than *B. pfeifferi* and remain active for longer time up to the eighth hour. Both species were not be able to attach firmly to the surface of petri dishes. Lymnaed snails seemed more irritated that they tried to crawl out of the solution. Snail mortalities in different concentrations and exposure times are presented in Tables 1 and 2.

**Table 1:** Mortalities of *B. pfeifferi* snails exposed to different concentrations of *A. aspera* aqueous leaf extract for 24, 48 and 72 h

Concentration (ppm)	24 h		48 h		72 h	
	Exposed	Dead	Exposed	Dead	Exposed	Dead
0	30	0	30	0	30	0
25	30	0	30	0	30	0
50	30	3	30	4	30	7
75	30	12	30	15	30	17
100	30	30	30	30	30	30
200	30	30	30	30	30	30
400	30	30	30	30	30	30

**Table 2:** Mortalities of *L. natalensis* snails exposed to different concentrations of *A. aspera* aqueous leaf extract for 24 h

Concentration (ppm)	Exposed snails	Dead snails	Mortality rate
0	30	0	0%
25	30	0	0%
50	30	3	10%
75	30	17	56.7%
100	30	29	93.3%
200	30	30	100%
400	30	30	100%

In addition to the negative controls made of only aged water, 0.28 ppm niclosamide solution was used as a positive control where, about 50% mortality rates were expected. The resulting mortalities were 56.7% (17 out of 30) and 53.3% (16 out of 30) for *B. pfeifferi* and *L. natalensis,* respectively, asserting to the normal experimental condition.

The data in Tables 1 and 2 showed that the plant has molluscicidal property. The noticeable concentration dependent mortalities of the two snail species ranged 25-100 ppm. Snail mortality in mid concentrations (50-75 ppm) also increased with prolonged exposure time. Analysis of effective doses, specifically LC_50_ and LC_90_ values that are useful to evaluate the plant’s molluscicidal efficacy are presented in Table 3.

**Table 3:** Molluscicidal effect of *A. aspera* aqueous leaf extract against *B. pfeifferi* and *L. natalensis* adult snails in terms of LC_50_ and LC_90_ values with 95% confidence limits (CL)

Snail species	Time	LC_50_ with CL	LC_90_ with CL	*χ* ^*2*^	Slope
*B. pfeifferi*	24 h	72.4 (66.5-78.1)	96.5 (88.0-112.6)	7.264	10.247
	48 h	69.9 (63.5-74.9)	93.8 (84.9-110.9)	5.063	9.650
	72 h	64.7 (58.5-70.7)	92.8 (82.9-112.7)	5.092	8.184
*L. natalensis*	24 h	69.5 (63.7-75.2)	93.9 (85.4-109.8)	1.021	9.824

**Fig. 1:**
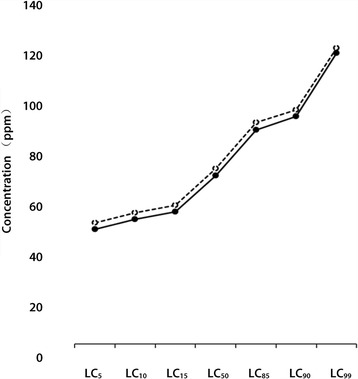
Relative sensitivities of *B. pfeifferi* and *L. natalensis* snails to various concentrations of *A. aspera* aqueous leaf extract*.* Broken line (-----) = *B. pfeifferi,* Solid line (**_____)** = *L. natalensis*

The calculated chi-square (*χ* ^*2*^) values in the analysis are less than the tabular values within the given degree of freedom indicating the mortality counts are not significantly heterogeneous and the goodness-of-fit of the probit model is acceptable. Moreover, the LC_50_ and LC_90_ values lie within the 95% confidence limits. As it can be seen from Table 3, for 24 h of exposure, the potency difference of the plant against the two snail species was relatively small. The graph below (Fig. 1) clarifies the trend along the entire LC levels. Yet, *B. pfeifferi* is somehow less sensitive than *L. natalensis* especially in concentrations below the LC_90_ level.

The phytochemical analysis of the aqueous leaf extract indicated presence of saponins and carbohydrates. However, tannins and other phenolics as well as flavonoids and alkaloids were absent. High saponin content is presumed from the vigorous emergence of persistent foam.

## Discussion

In Sub Saharan Africa, schistosomiasis is widely distributed especially in poor rural communities imposing huge socio-economic burden. Hence, community based low cost snail control measures are proved to be feasible. Furthermore, in relatively arid regions like Ethiopia where transmission is usually focal and seasonal, this self-help approach is preferable. Environmentally safe, easily applicable and locally available control agents like molluscicidal plants were recorded to be useful tools to enhance community participation [21]. The cost benefit advantage of such plant products over niclosamide is clearly stated in many literatures [22, 23]. *A. aspera* is an abundant wild herb in Ethiopia, easily growing on abandoned lands and roadsides. This plant is noncommercial and freely available to local communities without any cost related to transport, storage, expertise and custom. Generally, using such local materials has great economic role in import substitution, income generation to local growers and wasteland utilization.

This particular study showed that *A. aspera* has molluscicidal effect against *B. pfeifferi* and *L. natalensis* snails with the resulting LC_50_ of 72.4 and 69.5 ppm respectively in 24 h exposure. It is indeed confirmatory to the preliminary screening test we had done previously. In addition, the LC_90_ values are within the limit set by WHO for plant molluscicidal screening test in which the aqueous extract should be effective in less than 100 ppm [7, 21]. Some toxicological studies indicated the plant has no significant toxic effect in mice [24, 25].

The present phytochemical analysis showed presence of saponins and carbohydrates while tannins, flavonoids, and alkaloids were absent. To the contrary, phytochemical screening test in Bhosale et al. [26] has indicated presence of tannins, flavonoids and alkaloids. Such differences usually arise from variations in extraction techniques and/or parts of the plant used.

According to the present result, saponins are the principal molluscicidal compounds in the aqueous extract of this plant. The molluscicidal property of saponins is well documented in many studies. The main effect on animal cells is formation of complex with plasma and membrane cholesterols causing cell membrane damage [10].

The result shows that, in *B. pfeifferi* snails, as the exposure time extends from 24 to 72 h, the LC_50_ decreases from 72.4 to 64.7 ppm. The reason could be the active ingredient in the plant product is released slowly or it remains stable in action for longer time. Exposure to sub lethal levels may also have gradual effect on the snail survival.

The 24 h LC_90_ lethal dose against *B. pfeifferi* is 96.5 ppm. This result is nearly similar to the LC_90_ values (89.50 and 97.55 ppm) of mesocarp and whole fruits of *Balanites aegyptiaca* that was tested by Molla et al. [27]. However, *A. aspera* is found to be less potent than other studied plants. For instance, in the work of Kiros and colleagues [7], the aqueous extract of *Glinus lotoides* fruits produced LC_90_ of 56.96 ppm.

In the current study, the 24 h LC_90_ of the plant against *L. natalensis* was 93.9 ppm indicating higher efficacy on these snails when compared to plants such as *Balanites aegyptiaca* in Molla et al. [27].

This particular study also showed that the effective doses of the plant against the two snails differ only slightly. According to Utzinger and Tanner [28], the two snail species usually coexist in common habitats. Their similarity in sensitivity to this plant is a useful phenomenon that a single effective dose could be applied for the control of such mixed population altogether.

## Conclusions

This is the first evaluation of the molluscicidal effect of *A. aspera* against two medically important snail species in Ethiopia. The study indicated the aqueous extract is effective at acceptable concentration. The plant is widely available in most parts of Ethiopia and is a well-known traditional medicine. Therefore, this plant can play a role in community based snail control activities through further studies on different snail species and life stages. Effectiveness studies in the field condition and evaluation of toxic effects on non-target organisms are the researchers’ future prospects.

## Additional file

Additional file 1: Multilingual abstract in the five official working languages of the United Nations. (PDF 815 kb)
