# Supplementary material for: Molluscicidal effect of Achyranthes aspera L. (Amaranthaceae) aqueous extract on adult snails of Biomphalaria pfeifferi and Lymnaea natalensis
Source: Infect Dis Poverty. 2017 Oct 16;6:133. doi: 10.1186/s40249-017-0349-4 (PMC5641997; doi:10.1186/s40249-017-0349-4)
Supplement: Additional file 1: — Multilingual abstract in the five official working languages of the United Nations. (PDF 815 kb) [file 40249_2017_349_MOESM1_ESM.pdf]

Translation of the abstract into the five official working languages of the United Nations

أثر مبيدات الرخويات مائية الاستخراج من نبات م ضريس Achyranthes aspera L. (الفصيلة القطيفية) على القواقع البالغة لـBiomphalaria pfeifferi و Lymnaea natalensi

بيلايهون مانديفرو، سيد تيكو ميريتا، ينيب تاريكو، أرجاو أمبيلو

ملخص
خلفية: داء البلهارسيات المعوي الناجم عن البلهارسيا المانسونية هو مرض واسع الانتشار في معظم أجزاء المرتفعات الإثيوبية. مكافحة الحلزون هي استراتيجية رئيسية في مكافحة البلهارسيا. استخدام منتجات النباتية الرخويات أصبح مثيرا للاهتمام نظرا لسهولة الوصول إليها واستخدامها. ويهدف هذا البحث لتقييم تأثير الرخويات Achyranthes aspera على قواقع Biomphalaria pfeifferi و Lymnaea natalensis التي لها أهمية طبية وبيطرية كبيرة في إثيوبيا.
الأسلوب: تعرضت القواقع البالغةB. pfeifferi تركيزات مختلفة من مستخلص الأوراق المائي لنبات eraA. asp لمدة 24 و 48 و 72 ساعة. وبالمثل، تعرضت القواقع البالغة L. natalensis إلى المستخلص لمدة 24 ساعة. تم تحليل بيانات الوفيات باستخدام نموذج انحدار الاحتمال الطبيعي. تم تحليل محتوى النبات الكيميائي النباتي باستخدام طرق الفحص القياسية.
النتائج: ثبت تأثير النبات الرخويات على نوعي الحلزون. كانت قيم LC_{50} و LC_{90} مقابل L. natalensis كانت 69.5 و 93.9 جزء في المليون على التوالي. في 24، 48 و 72 ساعة من التعرض كانت قيمB. pfeifferi ، LC_{50} 72.4، 69.9، 64.7 جزء في المليون وLC_{90} 96.5، 93.8، 92.8جزء في المليون، على التوالي. وأظهرت اختبارات الفحص الكيميائي النباتي وجود الصابونين.
استنتاج: من نتائج هذه الدراسة، نجد أن لدىA. asperaالقدرة على إبادة الرخويات. وتوفر النتيجة أساسا مفيدا لإجراء مزيد من الدراسات المتعمقة لضمان قابليتها للتطبيق على نطاق أوسع في مختلف مسطحات المياه وتقييم آثارها السامة على الأنواع غير المستهدفة.

Translated from English version into Arabic by Mahmoud Sami, through

TRANSLATORS WITHOUT BORDERS

土牛膝（苋科）的水提取物对菲氏双脐螺（***Biomphalaria pfeifferi***）和纳塔尔椎实螺（***Lymnaea natalensis***）成年螺的灭螺效果

Belayhun Mandefro, Seid Tiku Mereta, Yinebeb Tariku, Argaw Ambelu

摘要
引言：由曼氏血吸虫造成的肠道血吸虫病在埃塞俄比亚高原大部分地区广泛肆虐。灭螺是血吸虫病防治的主要策略之一。植物灭螺产品因其易获取且实用，正逐渐引起人们的兴趣。本研究的目的在于评价土牛膝（*Achyranthes aspera*）对埃塞俄比亚具有重大医学和兽医学重要性的菲氏双脐螺和纳塔尔椎实螺的灭螺效果。
方法：成年菲氏双脐螺分别暴露于不同浓度的土牛膝叶水提取物 24、48 和 72 h。同样，成年纳塔尔椎实螺暴露于该提取物 24 h。使用概率回归模型分析死亡率数据。采用标准筛选法分析植物化学成分。

结果：该植物提取物对这两种螺的灭螺效果得到证实。暴露 24 h 后，纳塔尔椎实螺的半数致死量（LC_{50}）和 90%致死量（LC_{90}）分别为 69.5 和 93.9 μg/ml，暴露 24、48 和 72 h 后，菲氏双脐螺的 LC_{50} 分别为 7.4、69.9、64.7 μg/ml，LC_{90} 分别为 96.5、93.8、92.8 μg/ml。植物化学成分筛选试验表明该提取物有皂苷成分。
结论：从研究结果来看，土牛膝具有灭螺潜力。研究结果为进一步深入研究确定其在不同水体中的广泛适用性和评估其对非靶标物种的毒性作用奠定基础。

Translated from English version into Chinese by Zhou Guan, edited by Pin Yang

CHINESE CENTER FOR DISEASE CONTROL AND PREVENTION
NATIONAL INSTITUTE OF PARASITIC DISEASES

Effet molluscicide de l'extrait aqueux d'*Achyranthes aspera* L. (Amaranthaceae) sur les escargots adultes des espèces *Biomphalaria pfeifferi* et *Lymnaea natalensis*

Belayhun Mandefro, Seid Tiku Mereta, Yinebeb Tariku, Argaw Ambelu

RÉSUMÉ
Contexte: La schistosomiase intestinale causée par *Schistosoma mansoni* est une maladie très répandue dans la plupart des régions d'altitude d'Éthiopie. La régulation des populations d'escargots est une stratégie majeure de lutte contre la schistosomiase. L'utilisation de produits molluscicides d'origine végétale devient intéressante en raison de leur disponibilité et de leur facilité d'emploi. Cette recherche vise à évaluer l'effet molluscicide d'*Achyranthes aspera* sur les escargots *Biomphalaria pfeifferi* et *Lymnaea natalensis*, qui ont une grande importance en médecine humaine et vétérinaire en Éthiopie.
Méthode: Des sujets adultes de l'espèce *B. pfeifferi* ont été exposés à différentes concentrations d'extrait aqueux d'*A. aspera* pendant 24, 48 et 72 heures. Des sujets adultes de *L. natalensis* ont également été exposés à cet extrait pendant 24 heures. Les données de mortalité ont été analysées à l'aide d'un modèle de régression en probits. La teneur en composés phytochimiques de la plante a été analysée par les méthodes de tri standard.
Résultats: L'effet molluscicide de l'extrait végétal a été démontré sur les deux espèces d'escargot. La CL_{50} et la CL_{90} à 24 heures sur *L. natalensis* étaient respectivement de 69,5 et 93,9 ppm. Les valeurs de CL_{50} sur *B. pfeifferi* à 24, 48 et 72 heures étaient respectivement de 72,4, 69,9 et 64,7 ppm et la CL_{90} de 96,5, 93,8 et 92,8 ppm respectivement. Les tests de tri phytochimique ont indiqué la présence de saponines.
Conclusion: Les résultats de cette étude démontrent qu'*A. aspera* possède un potentiel molluscicide. Ils constituent une base utile pour d'autres études d'approfondissement qui vérifieront son applicabilité plus large à différents plans d'eau et évalueront ses effets toxiques sur les espèces non visées.

Translated from English version into French by Suzanne Assenat, through

TRANSLATORS
WITHOUT BORDERS

Моллюскоцидный эффект *водного раствора экстракта кадалади (Achyranthes aspera),* семейства амарантовых на взрослых особей улиток *Biomphalaria pfeifferi* и *Lymnaea natalensis*

Белахун Мандефро, Сеид Тику Мерета, Йинебеб Тарику, Арго Амбелу

Аннотация

Справочная информация: Кишечный шистосомоз, вызванный *шистосомой Мансони* это широко распространённое заболевание, охватывающее большинство районов Эфиопского нагорья. Регулирование популяции улиток является одной из основных стратегий управления шистосомозом. В связи с доступностью и простотой в применении возрастает интерес к использованию продуктов моллюскоцидных растений. Данное исследование направлено на оценку моллюскоцидного эффекта *кадалади (Achyranthes aspera)* на улиток*Biomphalaria pfeifferi* и *Lymnaea natalensis* и представляет огромную медицинскую и ветеринарную важность для Эфиопии.

Метод: взрослые улитки *B. pfeifferi* были подвержены воздействию различных концентраций*водного экстракта A. aspera*на 24, 48 и 72 часа. Аналогичным образом, взрослые особи улиток *L. natalensis* были подвержены воздействию экстракта на 24 часа. Данные о смертности были проанализированы с использованием пробит регрессионной модели. Анализ фитохимического состава растения был произведён с использованием методов стандартного скрининга.

Результаты: Был продемонстрирован моллюскоцидный эффект растения на двух особей улиток. Значения LC_{50} и LC_{90} при 24 часовом воздействии на *L. natalensis* составляли 69.5 и 93.9 ppm соответственно. За период 24, 48 и 72 часов воздействия на *B. pfeifferi*, значения LC_{50} составляли 72.4, 69,9, 64,7 ppm, а показатели LC_{90} соответствовали 96.5, 93,8, 92,8 ppm соответственно. Результаты фитохимического скрининга выявили присутствие сапонинов.

Заключение: Согласно результатам данного исследования, *A. aspera* обладает моллюскоцидным потенциалом. Результат создаёт полезную основу для дальнейших углубленных исследований с целью обеспечения более широкого применения укзанного растения в различных водоемах, а также оценки его токсических эффектов на нецелевые виды.

Translated from English version into Russian by Liudmila Tomanek (nee Volynets), through

TRANSLATORS
WITHOUT BORDERS

Efecto molusquicida del extracto acuoso de *Achyranthes aspera* L. (Amaranthaceae) sobre caracoles adultos de *Biomphalaria pfeifferi* y *stagnalis natalensis*

Belayhun Mandefro, Seid Tiku Mereta, Yinebeb Tariku, Argaw Ambelu

RESUMEN

Antecedentes: La esquistosomiasis intestinal, causada por *Schistosoma mansoni* es una enfermedad generalizada en la mayor parte de las tierras altas etíopes. El control del caracol es una importante estrategia en el control de la esquistosomiasis. El uso de productos molusquicidas vegetales resulta interesante debido a su accesibilidad y fácil aplicación. El objetivo de esta investigación es evaluar el efecto molusquicida de *Achyranthes aspera* sobre caracoles *Biomphalaria pfeifferi* y *Lymnaea natalensis* que son de gran importancia médica y veterinaria en Etiopía.

Método: Se expusieron caracoles adultos *B. pfeifferi* a diferentes concentraciones de extracto acuoso de hojas de *A. aspera* durante 24, 48 y 72 horas. De manera similar, caracoles adultos de *L. natalensis* se expusieron al extracto durante 24 horas. Los datos de mortalidad se analizaron con el modelo de regresión probit. Se analizó el contenido fitoquímico de la planta mediante el uso de métodos estándar de cribado.

Resultados: se demostró el efecto molusquicida de la planta sobre las dos especies de caracol. Los valores de CL_{50} y CL_{90} contra *L. natalensis* a las 24 horas fueron de 69,5 y 93.9 ppm respectivamente. A las 24, 48 y 72 horas de exposición de *B. pfeifferi*, los valores de CL_{50} fueron de 72,4, 69,9, 64,7 ppm y los de CL_{90} fueron de 96,5, 93,8, 92,8 ppm, respectivamente. Las pruebas fitoquímicas de cribado indicaron la presencia de saponinas.

Conclusión: con base en los resultados de este estudio, *A. aspera* tiene potencial molusquicida. El resultado proporciona una base útil para estudios posteriores de profundidad con el fin de garantizar su aplicación más amplia en distintos cuerpos de agua y evaluar sus efectos tóxicos sobre especies no objetivo.

Translated from English version into Spanish by Lidia Lariño, through

TRANSLATORS
WITHOUT BORDERS
